# Rush Medical College Commencement

**Published:** 1869-02-01

**Authors:** 


### *Rush Medical College Commencement.*

Postponement of the issue of the present number enables us to announce the occurrence of the Annual Commence- of Rush College, closing the most prosperous session it has ever enjoyed. In default of time we "scissor" the report made to the *Times:*

The 26th Annual Commencement of Rush Medical College took place in the lecture room of the institution, on the corner of Indiana and Dearborn streets.

The exercises were opened by a prayer by Rev. Dr. Erskine, after which the President of the College, Dr. J. V. Z. Blaney, delivered the usual charge to the class. The address abounded iu good advice to the young men who formed the graduating class. At the conclusion of his remarks he presented the diplomas to the graduating class.

THE GRADUATES.

The following are the names of the class: William H. Austin, James M. Adams, M. O. Baldwin, Russell Broughton, Thompson R. Brady, Frank L. Bradley, John W. Bacon, Hiram H. Bardwell, Samuel H. Birney, David J. Brookings, Robert Briggs, William M. Burton, Robert H. Brown, Simon P. Brown, Arthur B. Brackett, Gallaudet O. Bailey, James Baker, Cassidy Chenoweth, Thos. Crossgrove, William G. Cochran, Israel Cunkle, John J. Cameron, John P. Cloyd, Nelson H. Church, Amos A. Covalt, James G. Cameron, Mauritz B. Carleman, Joseph W. David, William A. Danforth, Wm. Dunlap, Michael Donnelly, Cyrenus A. David, Arthur W. Edwards, James H. Ethridge, Frank M. Elliott, John W. Firkins, Ezra K. Friermood, Gustav H. C. Fricke, Lee W. Fulton, James R. Fyffe, William A. Gordon, Oliver Gard, Joseph B. Galer, Job L. Gregory, Charles W. Goodale, James E. Groesbeck, Julius F. C. Hoffman, John B. Hamilton, Herbert S. Hill, William C. Hoover, Melancthon Hilbert, Charles E. Hogeboom, James R. Holgate. William C. Johnson, John M. Jenkins, Peter E. Kierland, Andres Klingberg, Joseph Knowles, Jahiel C. Kilgore, Frederic H. Linde, Justin J. Leavitt, Hugh E. Lindsay, George W. Lee, Jr., Augustus R. Logan, Joel W. Morris, Russell L. Moore, Stephen P. McClure, Adam E. Miller, Andrew J. Miller, John McGinnis, John C. Morgan, James M. McLean, Samuel McClellan, Jas. S. Moffatt, Wm. Monroe, Wmilliam F. Nichols, John E. O'Brien, Lorenzo Northrup, Almon Patterson, Thos. W. Parker, John B. Ralph, Robt. N. Rickey, Harley G. Ristine, George W. Roberts, Vincent H. Rose, George W. Stewart, Frank D. Stannard, Wm. H. Schrock, Alonzo B. Shepard, Byron N. Stevens, Fred. F. Sovereign, Chas. C. Sprague, Joshua B. Sprague, Thomas B. Spaulding, Albert R. Tucker, Dallas G. M. Front, Sylvester Thompson, James Tweddale, William L. Underwood, William H. Wirt, George H. Walker, Solon

C. White, Otto B. Will, Basil M. Webster, George Williamson, John Williamson, Frank S. Wadsworth, John L. Whitley.

### AD EUNDEM.

Frederick L. Matthews, M. D., of Illinois; Thomas R. McInnes, M. D., of Ontario, Canada; Robt. Tobey, M. D., of Ohio; John S. Clark, M. D., of Illinois.

### HONORARY.

Oliver Everett, M. D., of Illinois.

After the reception of the diplomas the address was responded to by Dr. Williamson, a graduate of the college, in a short address, which was well received by the audience.

### ADDRESS OF DR. ROSS.

Professor J. P. Ross delivered the valedictory address.

He gave a short but interesting account of the growth and progress of Rush Medical College from its foundation to the present time. He referred to the proud history of medicine in the world; to the long line of eminent men who have graced its ranks, and then proceeded to give the class some hints in regard to the means of making themselves most useful in the profession in which they were about to embark. He reminded them that they would find that the knowledge they had obtained was largely theoretical. They must perfect themselves by careful observation and practice New cases of study will present themselves. The cultivation of the senses to obtain the phenomena of disease, and the cultivation of the reasoning powers by which the facts presented through the senses may lead to a correct mode of the treatment of disease. To be successful they must determine to be students for life. This is the only way to become eminent. Without study a physician will soon find himself at the bottom of the therapeutical ladder. They must discard all inducements to neglect study. The speaker referred to a young man in the present class as a model of industry and perseverence, and predicted for the person, who remained anonymous, to most of the audience, that within ten years he would stand at the head of the profession he had adopted. Dr. Ross reverted to the revelations of science, and said they were developing the science of medicine with remarkable rapidity. In conclusion, the speaker urged upon his hearers the necessity of the adoption of correct moral principles. They should not make self-interest their great aim. At the bedside of the dying they should be able to offer the consolations of the purest religion.

The exercises closed with a benediction by Rev. Dr. Erskine.

---

MARRIED.—Feb. 1st, at the residence of the bride's father, Hon. D. Smiley, of Albany, Wis., by the Rev. G. R. Patton, of Inda, Wis., R. BROUGHTON, M. D., (of the class of 1869 at Rush), to MISS JULIA A. SMILEY, of the former place.

